# Prospective Study Investigating the Efficacy and Safety of a Scalp Cooling Device for the Prevention of Alopecia in Women Undergoing (Neo)Adjuvant Chemotherapy for Breast Cancer

**DOI:** 10.3390/curroncol29100569

**Published:** 2022-09-30

**Authors:** Luisa Carbognin, Cristina Accetta, Danilo Di Giorgio, Paola Fuso, Margherita Muratore, Giordana Tiberi, Francesco Pavese, Tatiana D’Angelo, Alessandra Fabi, Diana Giannarelli, Alba Di Leone, Stefano Magno, Giorgia Garganese, Alejandro Martin Sanchez, Daniela Andreina Terribile, Gianluca Franceschini, Riccardo Masetti, Giovanni Scambia, Ida Paris

**Affiliations:** 1 Department of Woman and Child Health and Public Health, Fondazione Policlinico Universitario Agostino Gemelli IRCCS, 00168 Roma, Italy; 2 Gynecology and Breast Care Center, Mater Olbia Hospital, 07026 Olbia, Italy; 3 Facility of Epidemiology and Biostatistics, Fondazione Policlinico Universitario Agostino Gemelli IRCCS, 00168 Roma, Italy; 4 Department of Woman and Child Health and Public Health, Università Cattolica del Sacro Cuore, 00168 Roma, Italy

**Keywords:** alopecia, breast cancer, chemotherapy, scalp cooling

## Abstract

The prevention of chemotherapy-induced alopecia still represents an urgent need for every day clinical practice. In this regard, this prospective single-center study included breast cancer (BC) patients who underwent a scalp cooling device (Dignicap^®^) during (neo)adjuvant chemotherapy with the aim to evaluate the efficacy and safety of this device in preventing alopecia. One hundred and seventy-eight patients (median age 43 years) were enrolled. The chemotherapy regimen included anthracycline and taxane-based chemotherapy (68.1%), docetaxel and cyclophosphamide (25.8%), anthracycline and taxane-based plus carboplatin (3.9%), and paclitaxel alone (2.2%). In 25.3% of cases, a dose dense schedule was used. Overall, the success rate was 68.0%: 100% in paclitaxel alone, 87.0% in docetaxel-cyclophosphamide, 59.5% in anthracycline and taxane, and 71.4% in the sequential regimen plus carboplatin group (anthracycline and taxane-based chemotherapy versus taxane-based chemotherapy, *p* ≤ 001. No difference in terms of hair preservation between dose-dense or standard schedule was found (*p* = 0.557). Early discontinuation of the scalp cooling was observed in 50 patients (28.1%). Although 138 patients (77.5%) experienced adverse events, 70.2% of patients were satisfied with this device. In conclusion, this large prospective study confirmed the helpful effect of the scalp cooling system in preventing alopecia in BC patients also undergoing sequential anthracyclines and taxane-based chemotherapy.

## 1. Introduction

Breast cancer (BC), accounting for approximately 30% of all new female cancer diagnoses in the United States and Europe, represents the most common cancer type in women [1,2]. Despite the recent efforts to spare the toxicity of antiblastics for the treatment of BC, chemotherapy remains a milestone for many patients affected by early-stage disease, corresponding to more than 90% of new cases [3]. Moreover, the increase in the dose intensity of adjuvant chemotherapy, by shortening the interval between treatment cycles, reduces the risk of recurrence and death from BC [4].

Chemotherapy-induced alopecia represents one of the most stressful side effects in patients undergoing neoadjuvant or adjuvant chemotherapy [5]. In order to prevent or reduce alopecia, a series of medical devices has been developed and tested. Most of these are based on scalp cooling systems that reduce the uptake of chemotherapeutic drugs in the hair follicles through a cutaneous vasoconstriction in the scalp [6]. The two scalp cooling devices mainly used worldwide are the DigniCap^®^ System (Lund, Sweden) and the Paxman^®^ System (Huddersfield, UK) [7]. These systems use a cap that is attached to a small, computer-controlled refrigeration machine that circulates cool liquid in the cap and controls the patient’s individual scalp temperature before, during, and after chemotherapy infusion [8]. A series of non-randomized and randomized trials have explored the role of scalp cooling systems in the prevention of alopecia in patients receiving chemotherapy for early-stage BC [9]. The successful rate in preventing alopecia (mainly defined as hair loss ≤ 50%) varies from 50% to 75%, according to different series [9]. However, the majority of patients included in these studies were undergoing anthracycline- or taxane-based chemotherapy regimens.

Recently, a prospective observational study including a subset of 63 patients underwent three-weekly anthracycline-based chemotherapy followed by weekly paclitaxel showed a 59% successful rate (defined as <50% hair loss not requiring a wig) [10]. Similar results, indicating a successful rate nearly 50%, were reported by subgroup analyses of prospective trials involving less than 50 patients treated with sequential anthracycline- and taxane-based chemotherapy [11].

Therefore, limited data concerning the efficacy of scalp cooling systems for sequential anthracycline- and taxane-based chemotherapy regimens are available. In addition, even less data are available for scalp cooling efficacy according to the chemotherapy schedule (dose-dense or standard).

In this regard, the aim of this study was to evaluate the efficacy and safety of a scalp cooling device in reducing chemotherapy-induced alopecia in women affected by BC receiving adjuvant or neoadjuvant treatment.

## 2. Materials and Methods

### 2.1. Study Design and Patient Population

This prospective observational single-center study conducted from July 2015 to June 2019 enrolled early BC patients undergoing neoadjuvant or adjuvant chemotherapy at the Oncological Gynecology Unit, Fondazione Policlinico Universitario Agostino Gemelli IRCCS of Rome. Inclusion criteria were: (1) Women aged 18 years or older; (2) early or locally advanced BC (stage I–III); and (3) eligibility to undergo scalp cooling during neoadjuvant or adjuvant chemotherapy administration.

### 2.2. Objectives

The primary aim of this study was to evaluate the efficacy of scalp cooling in the prevention of hair loss, according to the medical staff-assessed Dean alopecia scale [12], after the completion of all cycles of chemotherapy. Secondary aims included evaluating the efficacy of scalp cooling in the prevention of hair loss: (1) after the last use of scalp cooling according to medical staff assessment; (2) after the first chemotherapy cycle according to patient self-reported assessment; and (3) after the last chemotherapy cycle according to the patient self-reported assessment. Successful hair prevention was defined as medical staff-assessed or patient self-reported maximum Dean alopecia scale score of 2 or less (≤50% hair loss).

In addition, the study aimed to assess the tolerability of the scalp cooling system, patient-reported scalp cooling device side effects, and satisfaction.

### 2.3. Study Assessment and Intervention

#### 2.3.1. Hair Loss Evaluation

Alopecia assessments (according to Dean scale) were evaluated by medical-staff and patients before each chemotherapy cycle and after the last cycle. The degree of alopecia was prospectively collected in the clinical documentation by the oncology staff, whereas the patients’ self-assessment of alopecia was collected through questionnaires administered by the study oncology nurse. The questionnaires were also adopted to assess: (1) potential side effects such as headache, dizziness, feeling of chilliness, scalp and neck pain; and (2) satisfaction with the scalp cooling system at the end of treatment. Patient satisfaction was reported as a positive or negative personal experience with the use of the scalp cooling system.

#### 2.3.2. Scalp Cooling

The DigniCap^®^ Scalp Cooling System (Dignitana AB) was adopted. The cooling procedure started 30 min before the start of the chemotherapy infusion (in order to allow gradual cooling of the scalp to the desired temperature) and was maintained during the infusion and for 60–120 min after the end of treatment (post-cooling period). The duration of the post-cooling period was dependent on the specific chemotherapy type and schedule, according to the manufacturer’s recommendations. Scalp temperature was maintained at 3–5 °C, as the pre-set temperature of the coolant.

#### 2.3.3. Chemotherapy Treatments

Patients were treated with one of the following regimens: (1) anthracycline and taxane-based chemotherapy including epirubicin and cyclophosphamide for four cycles (q3w or q2w) followed by paclitaxel (q1w for 12 cycles or q2w for four cycles or q3w for four cycles) or epirubicin and cyclophosphamide for four cycles (q3w or q2w), followed by docetaxel for four cycles (q3w) or epirubicin and cyclophosphamide for four cycles (q3w or q2w), followed by weekly paclitaxel and carboplatin for 12 cycles; and (2) taxane-based chemotherapy including weekly paclitaxel for 12 cycles or docetaxel and cyclophosphamide for four cycles (q3w). Patients with HER2-positive disease also received trastuzumab. Dose reductions and delays were permitted as per the standard guidelines.

### 2.4. Statistical Analysis

Quantitative variables were summarized using the median and interquartile range while categorical variables were reported as the absolute frequencies and percentages. Comparisons among groups were performed with Pearson’s chi-squared (χ2) test or Fisher’s exact test, as appropriate. A logistic regression model was implemented with a univariate and multivariate approach to further assess the associations between alopecia grades and both the patient and treatment characteristics; odds ratios (OR) and 95% confidence intervals (CI) were reported. The level of significance was set at *p* ≤ 0.05. SPSS software (IBM SPSS statistics, v.27.0, Armonk, NY, USA) was used for statistical evaluations.

## 3. Results

### 3.1. Patient Characteristics

From July 2015 to June 2019, 178 patients with early or locally advanced BC stage underwent scalp cooling were enrolled in the study. The baseline patients’ characteristics are listed in Table 1. The median patient age at diagnosis was 43 years (interquartile range 37–47 years). The majority of patients were pre-menopausal at diagnosis (85.4%) and presented a HER2-negative disease (72.5%); 33 patients (18.5%) were treated for a triple negative disease. One hundred and four patients (58.4%) received chemotherapy in the adjuvant setting and 74 patients (41.6%) in the neoadjuvant setting.

With regard to treatments, the most common chemotherapy regimen was anthracycline and taxane-based chemotherapy (68.1%), followed by taxane-monotherapy based chemotherapy with docetaxel and cyclophosphamide (25.8%). The dose dense schedule was given in 25.3% of cases.

### 3.2. Scalp Cooling and Hair Loss

Overall, the success rate in the prevention of hair loss (Dean alopecia score 0–2), according to medical staff assessment, after the completion of all cycles of chemotherapy was 68.0%, meaning that 121 patients out of 178 experienced hair loss ≤ 50%.

The analysis of the secondary objectives of the study indicated that the success rate was: (1) 81.5% (145/178), after the last use of scalp cooling, according to medical staff assessment; (2) 61.2% (93/152) after the first chemotherapy cycle, according to patient self-reported assessment; and (3) 44.1% (64/145) after the last chemotherapy cycle, according to patient self-reported assessment.

Finally, excluding the 24 patients (13.5%) who discontinued the scalp cooling for reasons other than hair loss > 50%, the success rate in the prevention of hair loss, according to medical staff assessment, after the completion of all cycles of chemotherapy was 78.6% (121/154).

Associations between hair loss degree and patient characteristics, according to medical staff assessment after the completion of all cycles of chemotherapy, are reported in Table 2. No difference in terms of hair loss according to hair structure, thickness, and hair manipulation during treatment (i.e., waving, dyeing, coloring) was reported. Of interest, previous alopecia was not associated with a higher risk of hair loss.

Table 3 reports the associations between hair loss degree and treatment characteristics. Considering the chemotherapy regimen, the success rate after the completion of all treatment cycles was better in the taxane-based chemotherapy group (100% (5/5) in paclitaxel alone and 87.0% (40/46) in docetaxel-cyclophosphamide) compared with the anthracycline and taxane-based chemotherapy group (59.5% (72/127) anthracycline and taxane without carboplatin and 71.4% (5/7) in anthracycline and taxane with carboplatin), *p* < 0.001. However, the subgroup analysis for paclitaxel alone and carboplatin-based chemotherapy is limited by small numbers. A statistically significant difference between anthracycline and taxane-based chemotherapy versus the docetaxel and cyclophosphamide regimen was also observed (*p* < 0.001). No difference in terms of hair preservation between the dose-dense or standard schedule was found (*p* = 0.557).

Associations between hair loss degree and patient or treatment characteristics, according to medical staff assessment after the last use of scalp cooling, are reported in Table S1.

Finally, a univariate and multivariate logistic regression analysis of hair loss degree, evaluated by medical staff after the completion of all cycles of chemotherapy, according to the patient and treatment characteristics was reported (Table 4). At the multivariate analysis, the chemotherapy regimen was the only independent factor (anthracycline and taxane plus anthracycline and taxane with carboplatin vs. docetaxel-cyclophosphamide plus paclitaxel alone (OR: 0.17, 95% CI (0.04–0.67); *p* = 0.011). The area under curve using the predicted probability based on the logistic model was 0.66 (95% CI: 0.53–0.79).

### 3.3. Adverse Events and Patient Satisfaction

One hundred and thirty-eight patients (77.5%) experienced adverse events related to the scalp cooling system including headache, scalp and neck pain, feeling of chillness, dizziness, and scalp burn, as reported in Table 5.

One hundred and twenty-eight patients (71.9%) completed all planned chemotherapy cycles with a scalp cooling system; 50 patients (28.1%) discontinued scalp cooling early due to adverse events in 13.5% of cases (24 patients) and hair loss > 50% in 14.6% of cases (26 patients). Among the causes of discontinuation due to adverse events, the feeling of chillness was the most frequent (58.3% of cases).

One hundred and twenty-five patients (70.2%) were satisfied with the scalp cooling system at the end of treatment (positive personal experience), whereas only twenty-eight patients (15.7%) were unsatisfied (missing data in 25 patients). No difference in terms of missing data between the group of patients with hair loss ≤ 50% and hair loss > 50% (according to medical staff assessment, after the completion of all cycles of chemotherapy) was reported (17/121 patients (14.1%) and 8/57 patients (14.0%), respectively, *p* = 0.998). Finally, excluding patients without satisfaction questionnaires, the satisfaction rate was 97.1% among the 104 patients with hair loss ≤ 50% and 49.0% among the 49 patients with hair loss > 50%, *p* < 0.001.

## 4. Discussion

This prospective study suggests that scalp cooling is highly effective in preventing alopecia in patients receiving (neo)adjuvant anthracycline- and/or taxane-based chemotherapy for early BC. Indeed, our analysis reported an overall hair preservation rate of 68.0%, meaning that only 57 patients among 178 reported a hair loss > 50%. These results are in line with previous studies that showed a success rate of scalp cooling of around 60%, ranging from 30% to 90% due to the heterogeneity of these studies. In this regard, a recent meta-analysis including 27 studies (2202 patients) with general good-quality data reported a 61.0% effectiveness rate (95% CI, 55.0–67.0%).

Several factors including age, ethnicity, cooling techniques and schedule, scalp temperature, and chemotherapy characteristics were investigated as potential influencers of scalp cooling efficacy. However, while it is well-known that the type of chemotherapy drugs and regimens can influence the success rate of scalp cooling, limited data regarding the role of hair characteristics are available [13]. Our study suggests that hair thickness, structure, density, and manipulation during chemotherapy did not influence the hair preservation rate. In this regard, a series of studies reported that hair thickness, the main hair characteristic investigated, was not a significant factor associated with hair preservation [14,15,16], whereas another analysis suggested an inverse association [17].

Finally, previous alopecia such as alopecia caused by hormonal imbalance, medical conditions, or family history does not represent a risk factor for hair loss.

With regard to the impact of chemotherapy regimens in preventing alopecia, the literature data are contradictory given that a series of studies reported a higher success rate in taxane-based chemotherapy compared to anthracycline and taxane-based chemotherapy [10,18,19], whereas other studies reported no differences between patients with anthracycline-containing and anthracycline-free regimens [20]. In our analysis, hair preservation was achieved in 88.0% of patients in the taxane-based chemotherapy group compared to 60.2% in the anthracycline and taxane-based chemotherapy group (*p* < 0.001). Notably, the highest success rates were obtained in the paclitaxel alone group (100%) and the docetaxel-cyclophosphamide group (87.0%). Moreover, in the multivariate analysis, the chemotherapy regimen resulted in being an independent factor of hair loss: the scalp cooling system is more effective in preventing alopecia induced by taxane-based compared to anthracycline and taxane-based chemotherapy.

These results are consistent with a previous prospective study including 135 patients who underwent a DigniCap^®^ scalp cooling system during (neo)adjuvant chemotherapy that reported a higher success in the non-anthracycline compared to anthracycline and taxane-based regimens (71.0% versus 54.0%, respectively, *p* < 0.001), with a 100% success rate in the weekly paclitaxel alone group [18]. Consistently, another Italian analysis showed an overall success rate of 71.7%:100% in the paclitaxel and docetaxel-cyclophosphamide group and 61.8% in the sequential anthracycline and taxane-based group [21]. Interestingly, a recent prospective cohort study suggested that scalp cooling, used concomitantly with one course of paclitaxel, did not influence the pharmacokinetic parameters of paclitaxel, indicating that that scalp cooling seems to not reduce or increase the efficacy or toxicity of this cytotoxic drug [22].

The impact of dose-dense schedule such as 2-weekly schedules of anthracycline and taxane-based therapy in preventing alopecia with scalp cooling is undetermined, given that most studies included standard schedule chemotherapy such as 3-weekly schedules of anthracycline. In our analysis, no difference in terms of success rate according to dose-dense or standard schedule was reported (64.4% versus 69.2%, *p* = 0.557). Similarly, a recent single-center retrospective study reported a satisfactory hair prevention rate of 60.0% with dose-dense regimens; however, only 15 out of 80 patients included in this study received a dose-dense schedule [23]. This aspect is relevant for clinical practice given the important role of the chemotherapy dose dense schedule in increasing the BC outcome [4].

In the present study, the secondary objectives suggested a difference in the assessment of alopecia score by medical staff compared to patients, with a lower hair preservation rate reported by patients. With regard to this concern, previous studies have indicated a low concordance rate between patient self-reported and medical staff- (or nurse-) reported Dean’s alopecia score, with a potential underestimation of hair loss evaluated by health care personnel [11,16].

With regard to scalp cooling tolerability, more than 70% of patients experienced an adverse event such as headache, scalp and cervical pain, feeling of chillness, dizziness, and scalp burn. These results are in accordance with a previous trial including 79 patients randomized to scalp cooling (DigniCap^®^ system) or observation, which reported an 86.8% rate of device-related adverse events [20]. However, despite the high incidence of adverse events, the rate of scalp cooling discontinuation due to these effects was low (13.5%). Moreover, about 70% of patients reported a positive personal experience at the end of treatment, with a very high satisfaction rate among patients with hair loss ≤ 50%.

## 5. Conclusions

Despite the observational nature, this study represents the largest monocentric prospective analysis exploring potential factors associated with the DigniCap^®^ system efficacy in a ‘real-world’ context. Our results confirmed the potential positive effect of the scalp cooling system in preventing alopecia, with a fairly good safety profile, in BC patients undergoing (neo)adjuvant chemotherapy. However, further research is needed to increase the patients’ tolerance of the scalp cooling by reducing the distress and side effects of this system.

## Figures and Tables

**Table 1:** The baseline patients’ characteristics.

Variable	N. of Patients (%)
Menopausal state at diagnosis	
*Premenopausal*	152 (85.4)
*Postmenopausal*	26 (14.6)
cT/pT	
*1*	85 (47.8)
*2*	79 (44.4)
*3*	10 (5.6)
*4*	4 (2.2)
cN/pN	
*0*	83 (46.6)
*1*	70 (39.3)
*2*	9 (5.1)
*3*	6 (3.4)
*Unknown*	10 (5.6)
Histological Grade	
1	5 (2.8)
2	70 (33.7)
3	95 (53.4)
*Unknown*	18 (10.1)
Disease Stage	
*I*	56 (31.5)
*II*	98 (55.0)
*III*	24 (13.5)
HER2 Status	
*Positive*	49 (27.5)
*Negative*	129 (72.5)
Estrogen Receptor Status	
*Positive*	134 (75.3)
*Negative*	44 (24.7)
Progesterone Receptor Status	
*Positive*	117 (65.7)
*Negative*	61 (34.3)
Histology	
*Ductal*	152 (85.4)
*Lobular*	14 (7.9)
*Other*	12 (6.7)
Type of Surgery	
*Quadrantectomy/Lumpectomy*	113 (63.5)
*Mastectomy*	65 (36.5)
Chemotherapy Setting	
*Neoadjuvant*	74 (41.6)
*Adjuvant*	104 (58.4)
Chemotherapy Regimen	
*Anthracycline and Taxane-based*	121 (68.1)
*Anthracycline and Taxane-based plus Carboplatin*	7 (3.9)
*Docetaxel and Cyclophosphamide*	46 (25.8)
*Paclitaxel alone*	4 (2.2)
Dose Dense Schedule	
*Yes*	45 (25.3)
*No*	133 (74.7)
Taxane Schedule	
*Paclitaxel weekly*	40 (22.5)
*Paclitaxel q21*	33 (18.5)
*Paclitaxel q14*	37 (20.8)
*Docetaxel q21*	68 (38.2)
Trastuzumab	
*Yes*	48 (27.0)
*No*	216 (73.0)

N, number.

**Table 2:** The hair loss degree, evaluated by medical staff after the completion of all cycles of chemotherapy, according to the patient characteristics.

Variable	Hair Loss ≤ 50% (Dean Score 0–2) N. of Pts (%)	Hair Loss > 50% (Dean Score 3–4) N. of Pts (%)	*p-*Value
Scalp temperature throughout chemotherapy and post-cooling	106 (66.7)	53 (33.3)	0.296 ^
*3°*	61 (69.3)	27 (30.7)	
*4°*	34 (59.6)	23 (40.4)	
*5°*	11 (78.6)	3 (21.4)	
Hair thickness	103 (68.2)	48 (31.8)	0.736 ^
*Fine*	50 (70.4)	21 (29.6)	
*Medium*	32 (64.0)	18 (36.0)	
*Thick*	21 (70.0)	9 (30.0)	
Hair structure	96 (67.1)	47 (32.9)	0.471 ^
*Straight*	45 (72.6)	17 (27.4)	
*Wavy*	35 (63.6)	20 (36.4)	
*Curly*	16 (61.5)	10 (38.5)	
Hair density	103 (66.9)	51 (33.1)	0.774 ^
*Low*	7 (58.3)	5 (41.7)	
*Medium*	67 (68.4)	31 (31.6)	
*High*	29 (65.9)	15 (34.1)	
Previous alopecia	106 (66.2)	54 (33.8)	0.035 ^^
*Yes*	16 (88.9)	2 (11.0)	
*No*	90 (63.4)	52 (36.6)	
Hair manipulation during the chemotherapy period	102 (75.6)	33 (24.4)	0.101 ^^
*Yes*	20 (90.9)	2 (9.1)	
*No*	82 (72.6)	31 (27.4)	

N, number; Pts, patients; ^ *p*-value: Pearson’s chi-square test; ^^ *p*-value: Fisher’s exact test.

**Table 3:** The hair loss degree, evaluated by medical staff after the completion of all cycles of chemotherapy, according to the treatment characteristics.

Variable	Hair Loss ≤ 50% (Dean Score 0–2) N. of Pts (%)	Hair Loss > 50% (Dean Score 3–4) N. of Pts (%)	*p*-Value
Dose dense schedule	121 (68.0)	57 (32.0)	0.557 ^
*Yes*	29 (64.4)	16 (35.6)	
*No*	92 (69.2)	41 (30.8)	
Chemotherapy Regimen	121 (68.0)	57 (32.0)	<0.001 ^* <0.001 ^^#^
*Anthracycline and Taxane*	72 (59.5)	49 (40.5)	
*Anthracycline and Taxane plus Carboplatin*	5 (71.4)	2 (28.6)	
*Docetaxel and Cyclophosphamide*	40 (87.0)	6 (13.0)	
*Paclitaxel alone*	4 (100)	0 (0.0)	
Type of Taxane	121 (68.0)	57 (32.0)	0.089 ^
*Paclitaxel weekly*	25 (64.1)	14 (35.9)	
*Paclitaxel q2w*	24 (64.9)	13 (35.1)	
*Paclitaxel q3w*	18 (54.5)	15 (45.5)	
*Docetaxel q3w*	54 (78.3)	15 (21.7)	

N, number; Pts, patients; * anthracycline and taxane/anthracycline and taxane plus carboplatin-based chemotherapy versus docetaxel and cyclophosphamide and paclitaxel alone; ^#^ anthracycline and taxane-based chemotherapy versus docetaxel and cyclophosphamide; ^ *p*-value: Pearson’s chi-square test.

**Table 4:** The univariate and multivariate logistic regression analysis of hair loss degree, evaluated by medical staff after the completion of all cycles of chemotherapy, according to the patient and treatment characteristics.

Variable	Univariate OR (95% CI)	*p*-Value	Multivariable OR (95% CI)	*p-*Value
Scalp temperature throughout chemotherapy and post-cooling		0.430		0.417
*3°*	Ref.		Ref.	
*4–5°*	1.31 (0.67–2.53)		1.46 (0.58–3.65)	
Hair thickness		0.737		0.926
*Fine*	Ref.		Ref.	
*Medium*	1.34 (0.62–2.89)		1.07 (0.41–2.81)	
*Thick*	1.02 (0.40–2.59)		0.85 (0.24–3.05)	
Hair structure		0.473		0.425
*Straight*	Ref.		Ref.	
*Wavy*	1.51 (0.69–3.31)		1.64 (0.63–4.27)	
*Curly*	1.65 (0.63–4.35)		2.13 (0.63–7.21)	
Hair density		0.871		0.857
*Low–Medium*	Ref.		Ref.	
*High*	1.06 (0.51–2.23)		1.09 (0.42–2.86)	
Dose dense schedule		0.557		0.557
*No*	Ref.		Ref.	
*Yes*	1.24 (0.61–2.52)		1.76 (0.27–11.70)	
Chemotherapy Regimen	Ref.	0.001	Ref.	0.011
*Anthracycline and Taxane + Anthracycline and Taxane plus Carboplatin*				
*Docetaxel and Cyclophosphamide+ Paclitaxel alone*	0.21 (0.08–0.52)		0.17 (0.04–0.67)	
Type of Taxane		0.097		0.838
*Paclitaxel weekly*	Ref.		Ref.	
*Paclitaxel q2w*	0.97 (0.38–2.48)		0.52 (0.09–3.09)	
*Paclitaxel q3w*	1.49 (0.58–3.84)		1.03 (0.27–3.84)	
*Docetaxel q3w*	0.50 (0.21–1.18)		1.42 (0.37–5.49)	

OR, odds ratio; CI, confidence interval; Ref., reference category.

**Table 5:** The adverse events of the scalp cooling system.

Variable	N. of Patients (%)
Adverse Events	138 (77.5)
*Headache*	68 (49.3)
*Scalp and Neck Pain*	47 (34.1)
*Feeling of Chillness*	14 (10.1)
*Dizziness*	8 (5.8)
*Scalp Burn*	1 (0.7)
Adverse Events leading to Scalp Cooling Discontinuation	24 (13.5)
*Feeling of Chillness*	14 (58.3)
*Headache*	9 (37.5)
*Scalp and Neck Pain*	1 (4.2)

N, number.

## Data Availability

Medical records and follow-up data were collected on an electronic database, REDCap (electronic data acquisition tools) hosted at the Fondazione Policlinico Universitario A. Gemellli, IRCCS, Rome.

## Supplementary Materials

The following supporting information can be downloaded at: www.mdpi.com/article/10.3390/curroncol29100569/s1, Table S1. Hair loss degree, evaluated by medical staff after the last use of scalp cooling, according to the patient characteristics.
