# Supplementary material for: Prospective Study Investigating the Efficacy and Safety of a Scalp Cooling Device for the Prevention of Alopecia in Women Undergoing (Neo)Adjuvant Chemotherapy for Breast Cancer
Source: Curr Oncol. 2022 Sep 30;29(10):7218–28. doi: 10.3390/curroncol29100569 (PMC9600590; doi:10.3390/curroncol29100569)
Supplement: Supplementary file 1 [file curroncol-29-00569-s001.zip › curroncol-1917140-supplementary.pdf]

Table S1. Hair loss degree, evaluated by medical staff after the last use of scalp cooling, according to the patient characteristics.

**Variable**	**Hair loss ≤ 50% (Dean score 0-2) N. of Pts (%)**	**Hair loss > 50% (Dean score 3-4) N. of Pts (%)**	***p-value***
Scalp temperature throughout CT and post-cooling	128 (80.5)	31 (19.5)	*0.303^*
3°	72 (81.8)	16 (18.2)	
4°	43 (75.4)	14 (24.6)	
5°	13 (92.9)	1 (7.1)	
Hair thickness	123 (81.5)	28 (18.5)	*0.409^*
Fine	61 (85.9)	10 (14.1)	
Medium	39 (78)	11 (22)	
Thick	23 (76.7)	7 (23.3)	
Hair structure	116 (81.1)	27 (18.9)	*0.502^*
Straight	53 (85.5)	9 (14.5)	
Wavy	43 (78.2)	12 (21.8)	
Curly	20 (76.9)	6 (23.1)	
Hair density	124 (80)	30 (19.5)	*0.805^*
Low	10 (83.3)	2 (16.7)	
Medium	80 (81.6)	18 (18.4)	
High	34 (77.3)	10 (22.7)	
Previous alopecia	129 (80.6)	31 (19.4)	*0.201^^*
Yes	17 (94.4)	1 (5.6)	
No	112 (78.9)	30 (21.1)	
Hair treatment during CT period	115 (85.2)	20 (14.8)	*0.196^^*
Yes	21 (95.5)	1 (4.5)	
No	94 (83.2)	19 (16.8)	
Dose dense schedule	145 (81.5)	33 (18.5)	*0.771^*
Yes	36 (80)	9 (20)	
No	109 (82)	24 (18)	
CT Regimen	145 (81.5)	33 (18.5)	*0.067^** *0.093^#*
Anthracycline and Taxane	94 (77.7)	27 (22.3)	
Anthracycline and Taxane plus Carboplatin	6 (85.7)	1 (14.3)	
Docetaxel and Cyclophosphamide	41 (89.1)	5 (10.9)	
Paclitaxel alone	4 (100)	0 (0)	
Type of Taxane	145 (81.5)	33 (18.5)	*0.489^*
Paclitaxel weekly	31 (79.5)	8 (20.5)	
Paclitaxel q2w	29 (78.4)	8 (21.6)	
Paclitaxel q3w	25 (75.8)	8 (24.2)	
Docetaxel q3w	60 (87)	9 (13)	

Legend – Table S1: N, number; CT, chemotherapy; *anthracycline and taxane-/anthracycline and taxane plus carboplatin-based chemotherapy versus docetaxel and cyclophosphamide and paclitaxel alone; #anthracycline and taxane-based chemotherapy versus docetaxel and cyclophosphamide; ^*p-value*: Pearson's chi-square test; ^^*p-value*: Fisher's exact test.
